# Understanding the mechanistic links between neurodevelopmental traits and internalising problems: the mediating roles of intolerance of uncertainty and negative problem orientation

**DOI:** 10.1186/s12888-026-08082-1

**Published:** 2026-06-06

**Authors:** Luca D. Hargitai, Lucy H. Waldren, Ross Robinson, Lucy A. Livingston, Florence Y. N. Leung, Punit Shah

**Affiliations:** 1 Department of Psychology, University of Bath, Bath, UK; 2 Avon and Wiltshire Mental Health Partnership NHS Trust, Bath, UK; 3 Department of Psychology, Institute of Psychiatry, Psychology and Neuroscience, King’s College London, London, UK; 4 Neuroscience and Mental Health Innovation Institute, Cardiff University, Cardiff, UK; 5 School of Social Sciences, College of Health, Science and Society, University of the West of England, Bristol, UK

**Keywords:** Autism, Attention deficit hyperactivity disorder, Intolerance of uncertainty, Negative problem orientation, Internalising problems

## Abstract

**Background:**

Autism and attention deficit hyperactivity disorder (ADHD) are common neurodevelopmental conditions that have both consistently been linked to internalising problems like anxiety and depression. However, the mechanisms that underpin these associations are unclear, especially in adults. To fill this research gap, we used a trait approach to investigate two theoretically relevant dispositional characteristics that may explain these relationships: intolerance of uncertainty (IU) and negative problem orientation (NPO).

**Methods:**

We recruited a large (*N* = 504), nationally representative sample of adults from the United Kingdom using Prolific. Participants provided basic demographic information and completed self-report measures of ADHD traits, autistic traits, internalising (depression and anxiety) symptoms, IU and NPO. Using parallel mediation analyses, we examined the roles of IU and NPO in the associations of ADHD and autistic traits with internalising symptoms.

**Results:**

We found that, together, IU and NPO fully mediated the association between autistic traits and internalising symptoms whilst accounting for participants’ ADHD traits, age, sex and education level (standardised indirect effects: IU = 0.100, NPO = 0.139). Interestingly, these two dispositional characteristics only partially mediated the link between ADHD traits and internalising symptoms (standardised indirect effects: IU = 0.049, NPO = 0.128), suggesting that additional factors not captured by the mediation model may contribute to this positive relationship.

**Conclusions:**

We conclude that a person’s perception of their capacity to tolerate uncertain or ambiguous situations and their views on their (in)ability to effectively solve problems in their day-to-day lives may underpin the strong association between autism and internalising problems and, to a lesser extent, between ADHD and internalising problems.

**Supplementary Information:**

The online version contains supplementary material available at 10.1186/s12888-026-08082-1.

## Background

Autism spectrum disorder (hereafter autism) is a neurodevelopmental condition with a continuously increasing prevalence among adults in the United Kingdom (UK; [1]). It is characterised by difficulties with social interactions and restrictive or repetitive patterns of behaviour [2]. While recent research has started to focus on the strengths associated with this lifelong neurodevelopmental condition (e.g. [3–5]), autistic individuals continue to face many challenges in their day-to-day lives. For example, autistic adults have one of the highest rates of unemployment in the UK, with only 29% of them being in full- or part-time employment, compared to 54% of all disabled adults and 82% of non-disabled adults [6]. Research has also highlighted their difficulties in forming and maintaining relationships [7–9], which may also contribute to the lower levels of social support and reduced quality of life observed in autistic compared to neurotypical samples (e.g. [10, 11]). Moreover, studies spanning child, adolescent, and adult samples have linked autism to various mood disorders, several different anxiety disorders and even psychiatric conditions like schizophrenia [12–15]. Among these psychiatric conditions, anxiety and depression occur most frequently, with prevalence rates in autistic adults estimated to be as high as 42% and 37%, respectively [16]. These mental health difficulties can be pervasive and severely disrupt the day-to-day functioning of autistic individuals, which then leads to further reductions in quality of life [17–20].

Another neurodevelopmental condition that has been associated with poor life outcomes is attention deficit hyperactivity disorder (ADHD). It is observed in around 3% of adults globally [21] and is characterised by symptoms such as attentional difficulties, restlessness, impulsivity and hyperactivity [2]. As seen in the autistic population, individuals with ADHD also experience a variety of difficulties in their daily lives, including education- and employment-related challenges, as well as strained interpersonal relationships [22–25]. For example, Biederman and Faraone [26] note that individuals with ADHD are less likely to reach academic milestones beyond high school, are less frequently in full-time employment and typically have a lower household income than neurotypical adults, which may all contribute to the comparatively lower quality of life observed among adults with ADHD [27, 28]. Mental health problems also appear to be highly prevalent in this group, such that adults with ADHD are around five times more likely to experience any mood disorder, around four times more likely to experience any anxiety disorder, and approximately three times more likely to experience any substance use disorder in their lifetime than neurotypical adults [29]. Notably, individuals with ADHD who also have a co-occurring internalising problem like anxiety or depression tend to experience greater social difficulties than individuals without these concurrent conditions [30, 31]. At the same time, individuals with internalising problems who have co-occurring ADHD have worse overall outcomes than those without ADHD (e.g. [32, 33]) .

While there is a growing body of research suggesting that both autism and ADHD are associated with internalising problems, which then lead to further reductions in overall wellbeing and quality of life [34], their relative contributions to mental health problems are poorly understood, especially in adulthood (see [35, 36]). This may partly result from the large overlap in the symptom profiles of these two neurodevelopmental conditions [37–39], which can make these unique relationships difficult to disentangle. Additionally, it is only with the most recent revision of the Diagnostic and Statistical Manual of Mental Disorders [2] that an individual can receive concurrent autism and ADHD diagnoses, making it difficult to recruit sufficiently large and well-powered clinical samples to draw comparisons between the mental health outcomes of autism-only, ADHD-only and autism-ADHD groups.

To overcome these challenges, we recently adopted a trait-based approach to investigate the relative contributions of autism and ADHD to internalising problems [40]. This approach to studying neurodiversity involves looking at continuously distributed and measurable characteristics that are linked to specific genetic and environmental factors thought to underpin a given neurodevelopmental condition (see [41, 42]). Crucially, these dimensional neurodivergent traits may be more closely linked to the underlying biological processes that drive a given condition than the commonly used diagnostic categories (see [42–44]). More broadly, dimensional approaches to psychopathology provide valuable information that is lost when individuals are categorised into ‘diagnosis’ and ‘no diagnosis’ groups, and can often offer a better fit for highly heterogenous clinical data [41, 45, 46]. Thus, a trait-based neurodiversity approach allows researchers to meaningfully investigate the nature of neurodevelopmental conditions using large, general population samples who complete quantitative trait measures of autism (e.g. [47]), and ADHD (e.g. [48]). This methodological approach is also consistent with the conceptual shift towards a more transdiagnostic view of neurodevelopmental conditions that embraces the concept of “neurodiversity” and allows clinicians working with neurodivergent individuals to better understand their specific challenges and particular additional needs and thus offer more person-centred support (see [42, 49–51]).

In line with the broader research literature, our study indicated that both autistic and ADHD traits are uniquely associated with depression, anxiety and overall internalising problems (see [40]). Interestingly, across three different analyses, we showed that ADHD traits were a stronger and more important predictor of internalising problems than autistic traits. This same pattern of results was observed when depression and anxiety symptoms were investigated separately. In other words, measurable characteristics associated with ADHD likely lead to worse mental health outcomes than those associated with autism.

Building on this study, we propose that investigating the relationships among these conditions further could play a critical role in identifying potentially vulnerable groups that may benefit from additional mental health support. For example, novel statistical methods like network analysis, which is increasingly used in neurodevelopmental research (e.g. [39, 52, 53]), can help us better understand the extent to which individual internalising symptoms overlap with specific autistic and ADHD traits. Along these lines, to gain a deeper understanding of *why* neurodevelopmental conditions and internalising problems frequently co-occur, we suggest that it may be important to consider which transdiagnostic factors and cognitive sub-components of anxiety and depression may be driving the associations that are being observed at the behavioural level. The present study aims to focus on two candidate transdiagnostic processes that appear to be theoretically relevant to advancing this line of research and may serve as useful targets for clinical interventions.

The first factor that has been theorised to contribute to the relationship between neurodevelopmental and internalising symptoms is intolerance of uncertainty (IU). This is a dispositional characteristic that is centred on strong negative beliefs about uncertainty and about the potential consequences of a situation being ambiguous or unpredictable [54, 55]. When faced with such situations, individuals who are highly intolerant of uncertainty typically experience emotional distress and difficulties in day-to-day functioning [56]. While IU has primarily been discussed in the context of anxiety disorders and is thought to be a sub-component of anxiety, it also appears to be associated with mood disorders like depression [54, 57–60]. Importantly, recent studies employing self-report methods have suggested that IU may also be implicated in neurodevelopmental conditions like autism and ADHD [61–64]. Indeed, autistic individuals often demonstrate a strong preference for predictability and an increased sensitivity to uncertainty (e.g. [64–66]), while research in children and adolescents with ADHD has highlighted that predictability and routines can provide them with a sense of control and lead to improvements in both internalising and externalising symptoms (e.g. [67, 68]).

Another possible transdiagnostic factor that may underlie the relationship between neurodevelopmental traits and internalising symptoms is a tendency to feel helpless and pessimistic when faced with problems that appear threatening. This set of negative attitudes towards (social) problem solving is commonly conceptualised as negative problem orientation (NPO; [69]). NPO has been linked to various mental health conditions, including depression and anxiety [70–76]. Difficulties in social problem solving more broadly have also been associated with autism (e.g. [77, 78]), and ADHD (e.g. [79–81]). Indeed, recent research has strongly hinted at the mediating role of social problem solving in the relationship between neurodivergent traits – particularly those associated with autism – and anxious and depressive symptomatology [82–84]. However, the specific role of the NPO component of social problem solving within these relationships is unclear and has not yet been investigated.

Addressing these gaps in the literature, the current study used a nationally-representative adult sample from the UK to investigate the role of self-reported dispositional characteristics – namely IU and NPO – in the associations of autistic and ADHD traits with internalising symptoms. Drawing on previous research, we hypothesised that both IU and NPO would be significant mediators. For the first time in this field, we also explored whether these mediation effects were stronger for autistic traits or ADHD traits using parallel mediation analyses.

## Methods

### Transparency and openness

Ethical approval was granted by the University of Bath Psychology Research Ethics Committee (PREC: 19–025) and all participants gave written informed consent before taking part in the study. All study procedures were in accordance with the ethical standards of the institutional committee and with the 1964 Helsinki Declaration and its later amendments or comparable ethical standards. We report how we determined our sample size, all data exclusions, all manipulations, and all measures used in the study within the subsequent sections. The dataset and analysis code are openly available on the Open Science Framework (see: https://doi.org/10.17605/OSF.IO/NGJHX). The analyses reported in this study were exploratory and, therefore, not preregistered.

### Participants

We used the online participant recruitment platform, *Prolific*, to obtain a large general population sample of adults, representative of the UK in terms of age and sex distribution based on Office for National Statistics data [85]. An individual was eligible to participate if they were at least 18 years old, a fluent English speaker and a resident of the UK. Following data collection, participants who failed either of the two simple attention checks, which had been integrated into the survey, were excluded (*n* = 12). The final sample consisted of 504 participants (247 [49.01%] male; 257 [50.99%] female), aged 18–79 years (*M* = 45.03 years, *SD* = 15.41 years), with 262 (51.98%) holding a Bachelor’s degree or higher qualification, which is slightly higher than rates typically observed in the UK adult population [86]. Of the sample, 33.13% met the screening threshold for probable autism [47] and 18.25% met the screening threshold for probable ADHD [48] (for further information, please see the *Clinical Characteristics of the Sample* section of the Supplementary Materials). Based on Monte Carlo power analyses [87, 88], this sample size had at least 95% statistical power to detect indirect effects for two mediators in a parallel mediation analysis, given a small-to‐medium standardised coefficient of 0.175 for each path in the model (*α* = 0.05, 5000 replications).

### Materials and procedures

Participants completed a series of widely used and psychometrically validated self-report measures in a randomised order and then provided information about their age (years), sex at birth (male, female, neither male nor female) and education level between 0 (no qualifications) and 7 (PhD or equivalent), as measured by the International Standard Classification of Education [89]. No additional information was collected about clinical diagnoses (however, see the *Clinical Characteristics of the Sample* section of the Supplementary Materials). Simple attention checks (e.g., *“Please select ‘Slightly Agree’ to show that you are reading the questions.”*) were integrated into the survey to assess data quality without impacting the validity of the self-report measures [90].

### Autistic traits

The 28-item Short Autism Spectrum Quotient (AQ-Short; 47) was used to measure social and non-social autistic traits. Participants answered items using a 4-point Likert scale, which ranged from 1 (*‘definitely agree’*) to 4 (*‘definitely disagree’*). For any items where agreeing with the statement indicated characteristics of autism, the scoring was reversed. This generated a total score between 28 and 112 for each participant, with higher scores indicating more autistic traits. Importantly, Grove and colleagues [91] showed that the AQ-Short is suitable for use in studies that include participant sex in the modelling, as it is potentially less biased towards the male autism phenotype than other autism trait measures (see also [92]). In the present study, the AQ-Short had good inter-item reliability (*α* = 0.84; *ω* = 0.85).

### ADHD traits

The Adult ADHD Self-Report Scale (ASRS; 48) assessed participants’ ADHD traits. The scale has 18 items, which measure the frequency of symptoms related to inattention and hyperactivity/impulsivity over the preceding 6-month period. Participants responded to items on a 5-point Likert scale that ranged from 0 (*‘never’*) to 4 (*‘very often’*). This produced total scores between 0 and 72, with higher scores corresponding to more ADHD traits. This measure has been shown to have good construct validity, with Kessler and colleagues [48] noting a 96.2% classification accuracy for ADHD when compared to clinician ratings. In the present study, the ASRS had good inter-item reliability (*α* = 0.90; *ω* = 0.90).

### Intolerance of uncertainty

The 12-item short version of the Intolerance of Uncertainty Scale (IUS-12; [93]) was used to assess participants’ tendency to perceive uncertainty and ambiguity as distressing. For each questionnaire item, participants indicated the extent to which the statement was characteristic of them using a 5-point Likert scale ranging from 1 (*‘not at all characteristic’*) to 5 (*‘entirely characteristic’*). This resulted in a total score between 12 and 60, with higher scores indicating greater IU. Importantly, the IUS-12 has good convergent validity with the full-length IUS [93] but shows greater measurement invariance across white and black samples [94]. In this study, the IUS-12 had good inter-item reliability (*α* = 0.92; *ω* = 0.92).

### Negative problem orientation

The Negative Problem Orientation Questionnaire (NPOQ; 69) was used to assess participants’ attitudes and typical responses to problems they face. For each of the measure’s 12 items, participants used a 5-point Likert scale ranging from 1 (*‘not at all true for me’*) to 5 (*‘extremely true for me’*) to indicate the extent to which the statement reflected how they view and react to problems. This generated a total score between 12 and 60, with higher scores reflecting greater NPO. The NPOQ is reported to be a psychometrically sound measure of problem orientation, with good test-retest reliability and high convergent and discriminant validity against existing self-report measures of pessimism, depression, anxiety, and problem-solving ability [69]. In this study, the NPOQ had good inter-item reliability (*α* = 0.95; *ω* = 0.95).

### Anxiety symptoms

The 7-item Generalised Anxiety Disorder Scale (GAD-7; [95]) is a brief screening tool that measured the frequency with which participants experienced anxiety symptoms in the preceding 2-week period, using a 4-point Likert scale that ranged from 0 (*‘not at all’*) to 3 (*‘nearly every day’*). This produced total scores between 0 and 21, with higher scores corresponding to more severe anxiety. Spitzer and colleagues [95] showed that the GAD-7 had good convergent validity using the Beck Anxiety Inventory (*r* = .72) and the Symptom Checklist-90 (*r* = .74). The GAD-7 had good inter-item reliability in the present study (*α* = 0.93; *ω* = 0.93).

### Depression symptoms

The 9-item Depression Module of the Patient Health Questionnaire (PHQ-9; [96]) is a short screening measure that assessed the frequency of participants’ depression symptoms in the preceding 2-week period. Using a 4-point Likert scale ranging from 0 (*‘not at all’*) to 3 (*‘nearly every day’*), this measure generated total scores between 0 and 27, with higher scores reflecting more severe depression. Martin and colleagues [97] showed that the PHQ-9 was associated with the Brief Beck Depression Inventory (*r* = .73), demonstrating its convergent validity and, in the present study, this measure had good inter-item reliability (*α* = 0.92; *ω* = 0.92).

### Statistical analyses

Data were analysed using R (version 4.5.2 [98]; for the analysis code, see: https://doi.org/10.17605/OSF.IO/NGJHX). Parallel mediation analyses were conducted using the *lavaan* package (version 0.6.21; [99]) to investigate the potential mediating roles of IU and NPO in the relationships of autistic traits and ADHD traits with internalising problems. Separate models were run for autistic traits and ADHD traits, each accounting for participants’ age, sex and education level as well as the traits of the other condition. As anxiety- and depression symptoms were highly correlated (*r* = .81, *p* < .001), these variables were combined into a composite measure of internalising problems for the main analyses by standardising and averaging the total scores of the two measures (following [100, 101]; see the *Variable Dictionary* in the *Online Supplementary Materials* for more information on how this variable was calculated). Following the four-step approach outlined by Baron and Kenny [102], we first assessed the correlations among variables and tested for the prerequisite significant associations between the potential mediators (i.e., IU and NPO) and both the independent variable (i.e., autistic traits or ADHD traits) and the dependent variable (i.e., internalising problems) using a series of regression analyses. If these criteria were met, mediation analyses with 95% bias-corrected and accelerated bootstrap confidence intervals (10000 resamples) around the total, direct and indirect effects were conducted. Both unstandardised and standardised effects are reported in these parallel mediation analyses.

## Results

Descriptive statistics and correlations among variables of interest are presented in Table 1. Consistent with previous research, there was a positive correlation between autistic and ADHD traits (*r* = .30, *p* < .001). Both autistic and ADHD traits were also correlated with both IU (autistic traits: *r* = .51, *p* < .001; ADHD traits: *r* = .43, *p* < .001) and NPO (autistic traits: *r* = .40, *p* < .001; ADHD traits: *r* = .50, *p* < .001), as well as with internalising problems (autistic traits: *r* = .32, *p* < .001; ADHD traits: *r* = .58, *p* < .001). Importantly, both proposed mediators were also significantly correlated with internalising problems (IU: *r* = .55, *p* < .001; NPO: *r* = .64, *p* < .001). As shown in Table 2, the prerequisite associations between independent, dependent and mediating variables within regression analyses were also significant. Harman’s single-factor test demonstrates that the current measures do not show common method bias (single factor shared variance = 25.84%; but see [103]).

**Table 1:** Descriptive statistics and correlations between study variables

Measures	Mean (SD)	1	2	3	4	5	6	7	8	9
1. Autistic traits	65.69 (11.02)	–								
2. ADHD traits	26.10 (11.45)	0.30***	–							
3. IU	31.64 (10.02)	0.51***	0.43***	–						
4. NPO	26.84 (10.63)	0.40***	0.50***	0.62***	–					
5. Age (years)	45.03 (15.41)	− 0.04	− 0.23***	− 0.19***	− 0.31***	–				
6. Sex (F: M)	257:247	0.13**	− 0.09	0.01	− 0.06	0.01	–			
7. Education level	3.68 (1.90)	− 0.09*	− 0.03	− 0.10*	− 0.08	− 0.11*	0.03	–		
8. Anxiety symptoms	5.92 (5.40)	0.28***	0.56***	0.54***	0.62***	− 0.27***	− 0.09*	− 0.04	–	
9. Depression symptoms	7.14 (6.34)	0.33***	0.55***	0.49***	0.59***	− 0.27***	− 0.01	− 0.07	0.81***	–
10. Internalising problems	0.00 (0.95)	0.32***	0.58***	0.55***	0.64***	− 0.28***	− 0.05	− 0.06	0.95***	0.95***

Note. * *p* < .05, ** *p* < .01, *** *p* < .001. Sex is coded as 0 = Female, 1 = Male

**Table 2:** Prerequisite regression analyses

Predictor	B [95% CIs] ^a^	SE(B) ^a^	β	*p*	sr^2^	VIF
**Model 1: Internalising Problems** ^**b**^		*F*(5, 498) = 63.56, *p* < .001, *R*^*2*^ = 0.39				
Autistic traits	0.01 [0.01, 0.02]	0.00	0.17	< 0.001	0.02	1.14
ADHD traits	0.04 [0.03, 0.05]	0.00	0.49	< 0.001	0.20	1.18
Age	-0.01 [-0.01, -0.01]	0.00	-0.17	< 0.001	0.03	1.07
Sex ^**c**^	-0.06 [-0.19, 0.08]	0.07	-0.03	0.390	0.00	1.04
Education Level	-0.02 [-0.06, 0.01]	0.02	-0.05	0.193	0.00	1.03
**Model 2: Internalising Problems**		*F*(6, 497) = 70.81, *p* < .001, *R*^*2*^ = 0.46				
IU	0.03 [0.02, 0.04]	0.00	0.33	< 0.001	0.07	1.56
Autistic traits	0.00 [-0.00, 0.01]	0.00	0.03	0.478	0.00	1.41
ADHD traits	0.03 [0.03, 0.04]	0.00	0.40	< 0.001	0.12	1.30
Age	-0.01 [-0.01, -0.00]	0.00	-0.13	< 0.001	0.02	1.09
Sex ^**c**^	-0.05 [-0.17, 0.08]	0.06	-0.02	0.466	0.00	1.04
Education Level	-0.01 [-0.04, 0.02]	0.02	-0.02	0.488	0.00	1.03
**Model 3: Internalising Problems**		*F*(6, 497) = 84.68, *p* < .001, *R*^*2*^ = 0.51				
NPO	0.04 [0.03, 0.05]	0.00	0.43	< 0.001	0.12	1.58
Autistic traits	0.00 [-0.00, 0.01]	0.00	0.04	0.217	0.00	1.27
ADHD traits	0.03 [0.02, 0.03]	0.00	0.34	< 0.001	0.08	1.38
Age	-0.00 [-0.01, -0.00]	0.00	-0.07	0.033	0.00	1.16
Sex ^**c**^	-0.01 [-0.13, 0.12]	0.06	-0.00	0.897	0.00	1.04
Education Level	-0.01 [-0.04, 0.02]	0.02	-0.02	0.595	0.00	1.03
**Model 4: Internalising Problems**		*F*(7, 496) = 77.74, *p* < .001, *R*^*2*^ = 0.52				
IU	0.02 [0.01, 0.03]	0.01	0.18	< 0.001	0.02	1.92
NPO	0.03 [0.02, 0.04]	0.00	0.35	< 0.001	0.06	1.95
Autistic traits	-0.00 [-0.01, 0.01]	0.00	-0.01	0.797	0.00	1.43
ADHD traits	0.03 [0.02, 0.03]	0.00	0.32	< 0.001	0.07	1.40
Age	-0.00 [-0.01, 0.00]	0.00	-0.07	0.041	0.00	1.16
Sex ^**c**^	-0.01 [-0.13, 0.11]	0.06	-0.01	0.857	0.00	1.04
Education Level	-0.00 [-0.03, 0.03]	0.02	-0.01	0.755	0.00	1.04
**Model 5: Intolerance of Uncertainty**		*F*(5, 498) = 55.68, *p* < .001, *R*^*2*^ = 0.36				
Autistic traits	0.38 [0.31, 0.45]	0.04	0.42	< 0.001	0.15	1.14
ADHD traits	0.24 [0.16, 0.31]	0.04	0.27	< 0.001	0.06	1.18
Age	-0.08 [-0.13, -0.03]	0.03	-0.12	0.001	0.01	1.07
Sex ^**c**^	-0.37 [-1.83, 1.07]	0.75	-0.02	0.612	0.00	1.04
Education Level	-0.36 [-0.76, 0.01]	0.20	-0.07	0.058	0.00	1.03
**Model 6: Negative Problem Orientation**		*F*(5, 498) = 58.16, *p* < .001, *R*^*2*^ = 0.37				
Autistic traits	0.28 [0.20, 0.35]	0.04	0.29	< 0.001	0.07	1.14
ADHD traits	0.32 [0.26, 0.39]	0.03	0.35	< 0.001	0.10	1.18
Age	-0.16 [-0.21, -0.11]	0.02	-0.23	< 0.001	0.05	1.07
Sex ^**c**^	-1.31 [-2.80, 0.23]	0.78	-0.06	0.089	0.00	1.04
Education Level	-0.38 [-0.78, 0.01]	0.20	-0.07	0.060	0.00	1.03

Note

^a^ 95% bias-corrected and accelerated bootstrap confidence intervals (5000 resamples) for B and robust standard errors (HC3) are reported due to violations of homoscedasticity

^b^ Results of Model 1 previously reported in [40]

^c^ Sex is coded as 0 = Female, 1 = Male

**Fig. 1:**
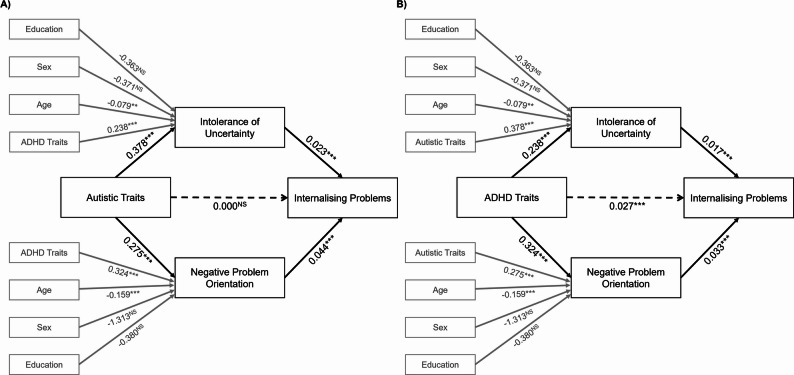
Parallel mediation models showing the unique mechanisms that drive the relationships of autistic and ADHD traits with internalising problems. *Note.* Panel **A** depicts the mediating roles of IU and NPO in the relationship between autistic traits and internalising problems, controlling for ADHD traits, age, sex and education level. Panel **B** depicts the mediating roles of IU and NPO in the association between ADHD traits and internalising problems, controlling for autistic traits, age, sex and education level. All coefficients are unstandardised. ***p < .001, **p < .01, *p < .05, ^NS^ non-significant

Parallel mediation analyses were, therefore, conducted to assess whether IU and NPO mediate the unique relationships of autistic traits and ADHD traits with internalising symptoms. The mediation models for autistic traits and ADHD traits are shown in Fig. 1A and B, respectively. The first parallel mediation analysis revealed a significant overall effect of autistic traits on internalising problems, controlling for ADHD traits, age, sex and education level (*total effect* = 0.021 [*SE* = 0.004, *95% bootstrap CIs*: 0.014–0.028], *β =* 0.244, *p* < .001). When accounting for the indirect paths via IU and NPO, the direct effect of autistic traits on internalising problems became non-significant (*direct effect* = 0.000 [*SE* = 0.003, *95% bootstrap CIs*: -0.006–0.007], *β* = 0.005, *p* = .891). When accounting for the direct path from autistic traits to internalising problems and the indirect path via NPO, there was a significant mediation via IU (*indirect effect via IU* = 0.009 [*SE* = 0.002, *95% bootstrap CIs*: 0.005–0.012], *β* = 0.100, *p* < .001). Specifically, individuals with more autistic traits tended to experience higher levels of IU (*b* = 0.378 [*SE* = 0.035, *95% bootstrap CIs*: 0.310–0.446], *β* = 0.415, *p* < .001) and being more intolerant of uncertain or ambiguous situations was linked to experiencing more internalising symptoms (*b* = 0.023 [*SE* = 0.004, *95% bootstrap CIs*: 0.014–0.031], *β* = 0.241, *p* < .001). There was also a significant mediation via NPO (indirect effect via NPO = 0.012 [SE = 0.002, 95% bootstrap CIs: 0.008–0.016], *β* = 0.139, *p* < .001) when accounting for the direct path and the indirect path via IU. Having more autistic traits was associated with more negative attitudes towards social problem solving (*b* = 0.275 [*SE* = 0.037, *95% bootstrap CIs*: 0.204–0.347], *β* = 0.285, *p* < .001), which in turn was linked to experiencing a greater number of internalising symptoms (*b* = 0.044 [*SE* = 0.004, *95% bootstrap CIs*: 0.036–0.051], *β* = 0.486, *p* < .001). Overall, these results suggest that the relationship between autistic traits and internalising problems is fully mediated by individual differences in self-reported IU and NPO.

The second parallel mediation analysis found a significant overall effect of ADHD traits on internalising problems, controlling for autistic traits, age, sex and education level (*total effect* = 0.041 [*SE* = 0.003, *95% bootstrap CIs*: 0.035–0.047], *β* = 0.499, *p* < .001). The direct effect of ADHD traits on internalising problems remained significant (*direct effect* = 0.027, [*SE* = 0.003, *95% bootstrap CIs*: 0.021–0.033], *β* = 0.322, *p* < .001) when accounting for the indirect paths via IU and NPO. There was a significant mediation via IU (*indirect effect via IU* = 0.004 [*SE* = 0.001, *95% bootstrap CIs*: 0.002–0.006], *β* = 0.049, *p* < .001) when accounting for the direct path from ADHD traits to internalising problems and the indirect path via NPO. That is, experiencing a greater number of ADHD traits was linked to higher levels of IU (*b* = 0.238 [*SE* = 0.034, *95% bootstrap CIs*: 0.172–0.305], *β* = 0.272, *p* < .001), which in turn was associated with more internalising symptoms (*b* = 0.017 [*SE* = 0.004, *95% bootstrap CIs*: 0.010–0.024], *β* = 0.179, *p* < .001). There was also a significant mediation via NPO (indirect effect via NPO = 0.011 [*SE* = 0.002, *95% bootstrap CIs*: 0.007–0.014], *β* = 0.128, *p* < .001) when accounting for all other paths in the model. Specifically, individuals with more ADHD traits tended to have higher levels of NPO (*b* = 0.324 [*SE* = 0.036, *95% bootstrap CIs*: 0.254–0.394], *β* = 0.349, *p* < .001), which was then associated with experiencing more internalising symptoms (*b* = 0.033 [*SE* = 0.004, *95% bootstrap CIs*: 0.026–0.040], *β* = 0.367, *p* < .001). Together, these results indicate partial mediation, such that ADHD traits were positively associated with internalising problems both directly and indirectly via IU and NPO.

## Discussion

This study aimed to gain insights into the transdiagnostic processes and unique mechanisms that may be driving the previously observed strong associations between neurodevelopmental conditions and poor mental health. Specifically, we investigated whether self-reported dispositional characteristics could help to explain the observed associations of autistic and ADHD traits with internalising symptoms. The analyses revealed that the positive relationship between autistic traits and internalising problems – that is, symptoms of anxiety and/or depression – was fully mediated by individual differences in IU and NPO. Previous research has suggested that a greater intolerance of uncertainty and negative attitudes towards one’s social problem-solving ability are both features of internalising problems and are also seen in autism (e.g. [59, 62, 69, 71, 75, 77, 83]) . Some studies have even shown that IU is a positive predictor of internalising symptoms in autistic samples (e.g. [63]). However, to our knowledge, this is the first study that has explored these dispositional characteristics together as potential mediators in the link between autism and internalising problems. Our findings highlight that individuals with higher levels of autistic traits not only view themselves as being less able to deal with uncertain or ambiguous situations but also tend to feel more helpless and pessimistic when faced with problems that appear threatening. These attitudes then appear to feed into heightened experiences of internalising problems. While it will be important to replicate these findings in clinical samples, they have potential implications for the mental health support that is offered to autistic individuals. Indeed, research has indicated that treatment as usual (e.g., standard behavioural activation for depression and worry management for anxiety; [104, 105]) may be less effective for the autistic than the general population (e.g. [106–109]) . Our results highlight that shifting the primary focus of therapy to challenging negative beliefs about uncertainty and working with autistic people to respond more constructively to the social problems they face in their daily lives could be particularly helpful in reducing their internalising symptoms. Crucially, as we controlled for ADHD traits in our analysis, our findings suggest that these mechanisms and arising clinical interventions may be robust in autistic people, irrespective of any co-occurring ADHD traits.

Our second parallel mediation analyses explored the association between ADHD traits and internalising problems. Mirroring the first analysis, individuals with more ADHD traits tended to perceive uncertainty as more distressing and endorsed more negative views about their problem-solving ability than those with fewer ADHD traits. High self-reported IU and NPO were then associated with experiencing more severe internalising problems. Interestingly, however, contrary to the analysis focusing on autistic traits, these dispositional characteristics only partially mediated the link between ADHD traits and internalising problems. This suggests that additional transdiagnostic factors, not captured in this mediation model, may contribute to this positive association. For example, ADHD may overlap with internalising problems at the cognitive level via difficulties with executive functioning. Research spanning the fields of psychology and neuroscience has highlighted that individuals with ADHD have difficulties in maintaining attention for sustained periods of time (e.g. [110, 111]), and inhibiting responses to irrelevant stimuli when attempting to complete a task (e.g. [112–115]). Although potentially under-investigated, difficulties with these executive function processes have also been observed in both depression (e.g. [116–118]), and anxiety (e.g. [119–121]). Equally, factors like emotion dysregulation [122–124] and alexithymia [125–127] may warrant further investigation.

The study reported here provides important and novel insights into potential mediators in the positive associations of autistic and ADHD traits with internalising problems among adults, a relatively understudied population within neurodevelopmental research [34–36, 128]. Crucially, we have shown, for the first time, that IU and NPO are important mechanisms underpinning the trait-level association between autism and internalising problems but may play a smaller role in the relationship between ADHD traits and internalising problems once autistic traits are taken into account. Our study also helps to strengthen the literature around the use of the trait-based approach in the conceptualisation of ADHD, which is relatively limited compared to its use within the autism literature [129, 130]. Nevertheless, further research is needed to examine whether our findings generalise to adults with clinically diagnosed autism and ADHD, and to address some methodological limitations of our study.

First, by focusing on only two candidate transdiagnostic processes, we may have neglected other, potentially relevant mediating factors (e.g., emotion dysregulation, alexithymia; [122, 126, 131, 132] that could help to explain some of the residual variance in the relationship between neurodevelopmental conditions and poor mental health. One way to address this remaining knowledge gap is to conduct a large-scale study examining the transdiagnostic profiles of autism, ADHD and internalising problems in adulthood, following similar research conducted in children [133–135].

Second, in considering only individual factors that could help to explain the frequently observed associations of autism and ADHD with internalising problems, we did not account for potentially relevant environmental influences. Indeed, the social model of disability [136, 137] highlights that environmental factors (e.g., physical surroundings, social network, stigma and victimisation) can have a notable impact on the wellbeing of neurodivergent adults [138–140]. Thus, an important next step in research will be to simultaneously investigate individual and environmental factors to understand their relative importance in explaining the link between neurodevelopmental conditions and poor mental health. This will help to identify the most important targets for intervention – both at the societal level (e.g., implementing specific modifications to make the environment more accessible, see [141]) and the individual level (e.g., adapting standardised therapy protocols to meet the specific needs of neurodivergent adults, see [108]) – to improve wellbeing in autism and ADHD.

Third, while the current study adds further cross-sectional evidence to the growing literature on the link between neurodevelopmental conditions and poor mental health, without longitudinal data, the direction of influence between neurodevelopmental traits, dispositional characteristics like IU and NPO, and internalising problems is not fully clear. Indeed, there is a large overlap in the symptoms of these different conditions [34, 142, 143], meaning that poor mental health could lead participants to self-report more traits associated with autism and ADHD. Thus, a well-powered longitudinal study is now needed to understand how these constructs interact over time and across the adult lifespan, and to make valid causal inferences about these relationships.

Finally, while we accounted for participants’ age, sex and education level within our analyses, we did not control for other potentially confounding demographic factors, such as their socioeconomic status or ethnicity, that have been linked to mental health within the literature (e.g. [144–146]) . Our study was also not designed nor able to account for genetic factors that are common across neurodevelopmental conditions and internalising problems (see [147]), which could have confounded the associations observed at the behavioural level. Thus, it will be important to investigate whether the mediation effects observed in our study are consistent across different ethnic groups, when participants’ socioeconomic status is considered and after accounting for shared genetic variance.

## Conclusions

Overall, our findings offer novel insights into the associations between neurodevelopmental traits and internalising problems. We showed that participants’ perception of their capacity to tolerate uncertain or ambiguous situations and their views on their (in)ability to effectively solve problems in their day-to-day lives explained the association between their autistic traits and internalising symptoms and, to a lesser extent, between their ADHD traits and internalising symptoms. Further research is now required to address the methodological limitations of this study and to gain a deeper, more comprehensive understanding of the psychological mechanisms that underpin the relationship between neurodevelopmental traits and internalising problems in adulthood.

## Supplementary Information

Below is the link to the electronic supplementary material.

Supplementary Material 1

## Data Availability

The dataset analysed during the current study and the analysis code used are openly available as *Online Supplementary Materials* on the Open Science Framework (see: https://doi.org/10.17605/OSF.IO/NGJHX).

## Abbreviations

ADHD: Attention Deficit Hyperactivity Disorder
AQ-Short: 28-item Short Autism Spectrum Quotient
ASRS: Adult ADHD Self-Report Scale
GAD-7: 7-item Generalised Anxiety Disorder Scale
IU: Intolerance of Uncertainty
IUS-12: 12-item short version of the Intolerance of Uncertainty Scale
NPO: Negative Problem Orientation
NPOQ: Negative Problem Orientation Questionnaire
PHQ-9: 9-item Depression Module of the Patient Health Questionnaire
UK: United Kingdom
