# Supplementary material for: Understanding the mechanistic links between neurodevelopmental traits and internalising problems: the mediating roles of intolerance of uncertainty and negative problem orientation
Source: BMC Psychiatry. 2026 Jun 6;26:628. doi: 10.1186/s12888-026-08082-1 (PMC13474798; doi:10.1186/s12888-026-08082-1)
Supplement: Supplementary file 1 [file 12888_2026_8082_MOESM1_ESM.docx]

**SUPPLEMENTARY MATERIALS**

*Understanding the mechanistic links between neurodevelopmental traits and internalising problems: the mediating roles of intolerance of uncertainty and negative problem orientation.*

**OPEN ACCESS DATA AND ANALYSIS CODE**

For the data, variable dictionary, analysis script and information about the R packages used for the analysis, please see: <https://doi.org/10.17605/OSF.IO/NGJHX>

**CLINICAL CHARACTERISTICS OF THE SAMPLE**

To better understand the clinical characteristics of our general population sample, we examined the distribution of neurodevelopmental traits across the participants. As shown in *Figures S1A* and *S1B*, both autistic traits and ADHD traits were approximately normally distributed in our sample, in line with expectations (see 1,2). We also explored the distribution of both anxiety and depression symptoms in our sample (see *Figures S1C* and *S1D*, respectively) and found that both distributions were positively skewed, consistent with previous research (see 3–5).

**Figure S1**

*The distribution of neurodevelopmental traits and internalising symptoms across the study sample.*

*Note.* Panel **A** depicts the approximately normal distribution of autistic traits in the sample, as measured by participants’ total scores on the AQ-Short (6). Panel **B** depicts the approximately normal distribution of ADHD traits in the sample, as measured by participants’ total scores on the ASRS (7). Panel **C** shows the positively skewed distribution of anxiety symptoms in the sample, as measured by participants’ total scores on the GAD-7 (8). Panel **D** shows the positively skewed distribution of depression symptoms in the sample, as measured by participants’ total scores on the PHQ-9 (9).

While we did not collect information about participants’ clinical diagnoses, the self-report measures used in our study to assess their neurodevelopmental traits and internalising symptoms can be used as screening tools. We used a cutoff score of ≥70 on the AQ-Short to categorise participants as potentially autistic versus non-autistic (see 6) and found that 167 (33.13%) of the participants met this threshold. To screen for probable ADHD, we used the first six items of the ASRS. Following Kessler et al. (7), ratings of 2 (*‘sometimes’*) or above on items 1-3 and ratings of 3 (*‘often’*) or above on items 4-6 were taken to indicate endorsement of a given symptom. Endorsing at least 4 of the 6 symptoms on the screener was used as a threshold for probable ADHD, and, in total, 92 (18.25%) of the participants met this threshold. We used a cutoff score of ≥8 on the GAD-7 to identify probable cases of generalised anxiety disorder (10). We found that 155 (30.75%) of the participants met the threshold for probable generalised anxiety disorder. Using a cutoff score of ≥10 on the PHQ-9 (11,12), we found that 145 (28.77%) of the participants met the threshold for probable depression.

**REFERENCES**

1.	Coghill D, Sonuga-Barke EJS. Annual Research Review: Categories versus dimensions in the classification and conceptualisation of child and adolescent mental disorders – implications of recent empirical study. Journal of Child Psychology and Psychiatry. 2012;53(5):469–89.

2.	Lyall K. What are quantitative traits and how can they be used in autism research? Autism Research. 2023;16(7):1289–98.

3.	Ferr H. The normal distribution is not normal in psychological data: Moving beyond parametric dogma. PLOS Mental Health. 2025 Aug 12;2(8):e0000403.

4.	Tomitaka S, Kawasaki Y, Ide K, Akutagawa M, Ono Y, Furukawa TA. Stability of the Distribution of Patient Health Questionnaire-9 Scores Against Age in the General Population: Data From the National Health and Nutrition Examination Survey. Front Psychiatry. 2018 Aug 23;9:390.

5.	Tomitaka S, Furukawa TA. The GAD-7 and the PHQ-8 exhibit the same mathematical pattern of item responses in the general population: analysis of data from the National Health Interview Survey. BMC Psychol. 2021 Sep 23;9(1):149.

6.	Hoekstra RA, Vinkhuyzen AAE, Wheelwright S, Bartels M, Boomsma DI, Baron-Cohen S, et al. The construction and validation of an abridged version of the autism-spectrum quotient (AQ-Short). J Autism Dev Disord. 2011 May;41(5):589–96.

7.	Kessler RC, Adler L, Ames M, Demler O, Faraone S, Hiripi E, et al. The World Health Organization adult ADHD self-report scale (ASRS): a short screening scale for use in the general population. Psychological Medicine. 2005 Feb;35(2):245–56.

8.	Spitzer RL, Kroenke K, Williams JBW, Löwe B. A Brief Measure for Assessing Generalized Anxiety Disorder: The GAD-7. Archives of Internal Medicine. 2006 May 22;166(10):1092–7.

9.	Kroenke K, Spitzer RL, Williams JBW. The PHQ-9: Validity of a Brief Depression Severity Measure. J Gen Intern Med. 2001 Sep 1;16(9):606–13.

10.	Plummer F, Manea L, Trepel D, McMillan D. Screening for anxiety disorders with the GAD-7 and GAD-2: a systematic review and diagnostic metaanalysis. General Hospital Psychiatry. 2016 Mar 1;39:24–31.

11.	Levis B, Benedetti A, Thombs BD. Accuracy of Patient Health Questionnaire-9 (PHQ-9) for screening to detect major depression: individual participant data meta-analysis. BMJ. 2019 Apr 9;365:l1476.

12.	Manea L, Gilbody S, McMillan D. Optimal cut-off score for diagnosing depression with the Patient Health Questionnaire (PHQ-9): a meta-analysis. CMAJ. 2012 Feb 21;184(3):E191–6.
